# Discovery of Antimycin-Type Depsipeptides from a *wbl* Gene Mutant Strain of Deepsea-Derived *Streptomyces somaliensis* SCSIO ZH66 and Their Effects on Pro-inflammatory Cytokine Production

**DOI:** 10.3389/fmicb.2017.00678

**Published:** 2017-04-19

**Authors:** Huayue Li, Huiming Huang, Lukuan Hou, Jianhua Ju, Wenli Li

**Affiliations:** ^1^Key Laboratory of Marine Drugs, Ministry of Education, School of Medicine and Pharmacy, Ocean University of ChinaQingdao, China; ^2^CAS Key Laboratory of Marine Bio-resources Sustainable Utilization, Guangdong Key Laboratory of Marine Materia Medica, RNAM Center for Marine Microbiology, South China Sea Institute of Oceanology, Chinese Academy of SciencesGuangzhou, China; ^3^Laboratory for Marine Drugs and Bioproducts of Qingdao National Laboratory for Marine Science and TechnologyQingdao, China

**Keywords:** antimycin-type depsipeptides, deepsea-derived bacteria, regulatory gene, anti-inflammatory, IL-5 production

## Abstract

Deepsea microbes are a rich source of novel bioactive compounds, which have developed unique genetic systems as well as biosynthetic pathways compared with those of terrestrial microbes in order to survive in extreme living environment. However, a large variety of deepsea-microbial secondary metabolic pathways remain “cryptic” under the normal laboratory conditions. Manipulation of global regulators is one of the effective approaches for triggering the production of cryptic secondary metabolites. In this study, by combination of various chromatographic purification process, we obtained somalimycin (**1**), a new antimycin-type depsipeptide, with an unusual substitution of 3-aminosalicylate instead of conserved 3-formamidosalicylate moiety, along with two known (**2** and **3**) analogs from the Δ*wblA_so_* mutant strain of deepsea-derived *Streptomyces somaliensis* SCSIO ZH66. The structures of **1–3** were elucidated on the basis of extensive spectroscopic analyses including LC-MS and NMR. In the evaluation of potent anti-inflammatory activity, compound **2** exhibited strong inhibitory activity on the IL-5 production in ovalbumin-stimulated splenocytes with IC_50_ value of 0.57 μM, while **1** and **3** displayed mild effect (>10 μM), which might be attributed to their different side-chain substitutions. Moreover, compounds **1–3** showed very weak cytotoxicity against human umbilical vein endothelial cells with LD_50_ values of 62.6, 34.6, and 192.9 μM, respectively, which were far over their IL-5 inhibitory activity. These results indicated that these compounds have good potential for further use in anti-inflammatory drug development.

## Introduction

Marine natural products are a rich source of drug candidates, an increasing number of which have been approved to the market or are currently in clinical trials (Newman and Cragg, 2004; Salomon et al., 2004; Kollar et al., 2014; Rangel and Falkenberg, 2015). In the expanding search for sources of new chemical and bioactivity diversity, the exploration of deepsea microbes has emerged as a new frontier in drug discovery and development. Deepsea microbes live in a biologically competitive environment with unique conditions of temperature, pressure, oxygen, light, salinity, and nutrients. In the long process of evolution, deepsea microbes developed unique genetic systems as well as different biosynthetic pathways compared with those of terrestrial microbes for good adaption to extreme living environment, which enables them to produce a large diversity of structurally novel and biologically active secondary metabolites (Skropeta, 2008; Skropeta and Wei, 2014).

Historically, the exploration of novel bioactive compounds was mostly “grind and find” mode, which frequently get half the result with twice the effort. The microbial secondary metabolites that have been reported by traditional methods are just the tip of the ice berg. A large variety of the biosynthetic pathways still remain “cryptic” under the normal laboratory conditions. With the recent advances in genomics and bioinformatics analytical techniques, genome-guided compound mining, the key point of which is to find ways to turn on or turn up the expression of cryptic or poorly expressed pathways to trigger novel compound production, has been used as a more efficient approach compared to those traditional methods for novel bioactive compound discovery (Challis, 2008; Lane and Moore, 2011; Bachmann et al., 2014).

Manipulation of global regulators is one of the effective strategies for triggering the production of cryptic secondary metabolites (Baltz, 2016). In our previous study, we inactivated the negative global regulatory gene *wblA_so_* in deepsea-derived *Streptomyces somaliensis* SCSIO ZH66, and it led to significant changes of secondary metabolites production in the Δ*wblA_so_* mutant strain, from which a series of anti-MRSA (methicillin-resistant *Staphylococcus aureus*) α-pyrone compounds (violapyrones A-C, H, and J) were isolated and identified (Huang et al., 2016).

**FIGURE 1:**
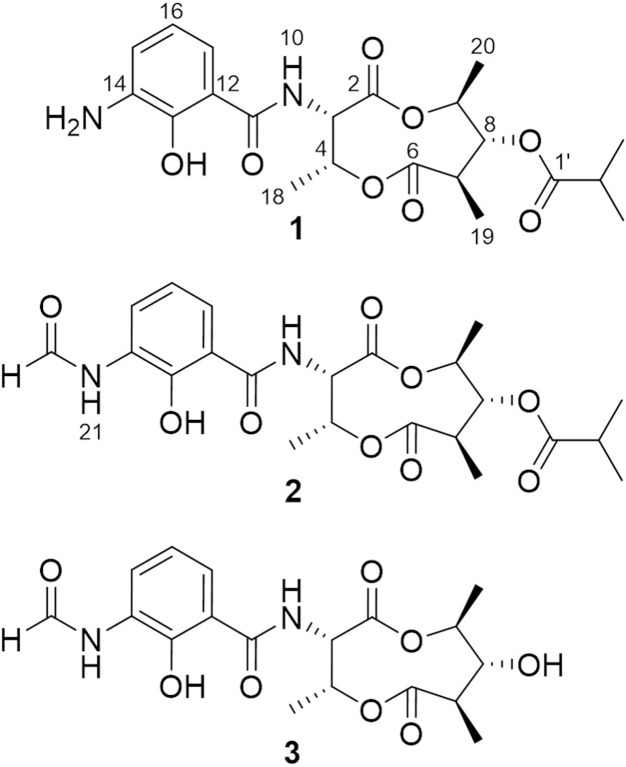
**Structures of compounds **1–3****.

In the continuing study of the same mutant strain, we found that, in addition to violapyrones, the production of several other secondary metabolites were notably increased as well compared to those in the wild-type strain. With the subsequent UV-spectrum-guided separation, we obtained a new antimycin-type depsipeptide, namely somalimycin (**1**), and two known analogs, USF-19A (**2**) and urauchimycin D (**3**) (**Figure 1**). The current paper deals with the isolation, structural elucidation, and biological evaluation of the compounds from the Δ*wblA_so_* mutant strain of deepsea-derived *S. somaliensis* SCSIO ZH66. Moreover, on the inhibitory effect of IL-5 cytokine production, the structure–activity relationship (SAR) study of compounds **1–3** with reported antimycin analogs were also performed.

## Materials and Methods

### Bacterial Strains and Culture Conditions

The *S. somaliensis* SCSIO ZH66 (CGMCC NO.9492) wild-type strain was isolated from the deepsea sediment collected at a depth of 3536 m of the South China Sea (120° 0.250′E; 20° 22.971′N; Zhang et al., 2015). The Δ*wblA_so_* mutant strain was constructed in our previous study (Huang et al., 2016). The strains were grown at 30°C on MS medium for sporulation, and were fermented as previously described (Huang et al., 2016).

### Isolation and Purification

**FIGURE 2:**
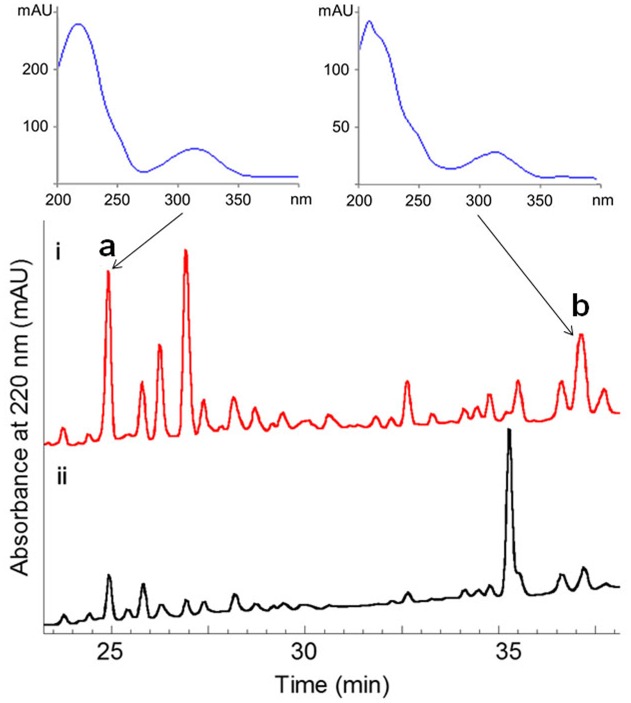
**Comparative HPLC analysis of secondary metabolites in the culture extracts of wild-type *S. somaliensis* SCSIO ZH66 and its Δ*wblAso* mutant strain.** The notably enhanced production displaying similar UV-spectra in the Δ*wblAso* mutant strain were marked with a and b. Compounds **1** and **2** were purified from peak b, and compound **3** was obtained from the enhanced peak a.

The fermentation broth (50 mL) of the wild-type and the Δ*wblA_so_* mutant strains was extracted with EtOAc, respectively, and was subsequently subjected to HPLC analysis (Agilent 1260 Infinity equipment). Analytical HPLC was performed with a linear gradient from 10 to 100% B/A in 50 min (mobile phase A: H_2_O + 0.1% HCOOH; phase B: 100% MeOH + 0.1% HCOOH; YMC-Pack ODS-A column 150 mm × 4.6 mm, i.d. 5 μm; wavelength: 220 nm) to analyze the production changes between the wild-type and mutant strains (**Figure 2**). The combined culture broth of the Δ*wblA_so_* mutant strain (20 L) was extracted with EtOAc at room temperature, which was partitioned between 90% MeOH and *n*-hexane to remove non-polar components. Then the MeOH layer was subjected to a stepped-gradient open column (ODS-A, 120 Å, S-30/50 mesh) eluting with 20–100% MeOH to yield five fractions. Each fraction was subjected to the HPLC to confirm the notably enhanced products in the mutant strain by their retention time and UV spectra. Compounds **1** (5.1 mg) and **2** (1.8 mg) were obtained by further purification of the enhanced peak **b** in fraction 4 on a reversed-phase HPLC (YMC-Pack ODS-A column 250 mm × 10 mm, i.d. 5 μm; wavelength: 220 nm) eluting with 85% MeOH + 0.2% HCOOH (v/v) (1 mL/min). Compound **3** (6.5 mg) was obtained from the enhanced peak **a** in fraction 3 eluting with 75% MeOH + 0.2% HCOOH (v/v) (1 mL/min). The structures of compounds **1–3** are shown in **Figure 1**.

*Somalimycin (**1**)*: dark brown, amorphous solid; CD (*c* 1.15 × 10^-3^ M, MeOH) *λ*_max_ (Δ*ε*) 228 (1.23), 245.5 (0.63), 262.5 (1.35) nm, 350 (-0.24) nm (Supplementary Figures S2, S3); ^1^H and ^13^C NMR data, see **Table 1**; HR-ESIMS *m/z* 437.1931 [M + H]^+^ (calcd for C_21_H_29_O_8_N_2_, 437.1918).

**Table 1:** ^1^H and ^13^C NMR chemical shifts of **1** in DMSO-*d*_6_.

	1	
	
Position	δ_H_ (*J* in Hz)	δ_C_
2		169.9
3	5.28 (1H, t, 7.8)	54.1
4	5.54 (1H, quin, 6.6)	71.8
6		174.5
7	2.65 (1H, m)	43.5
8	4.83 (1H, t, 10.2)	76.3
9	4.92 (1H, m)	73.9
10-NH	9.26 (1H, d, 8.4)	
11		170.2
12		115.0
13		151.4
14		129.1
15	7.20 (1H, d, 7.8)	123.7
16	6.87 (1H, t, 7.8)	119.1
17	7.74 (1H, d, 7.8)	122.4
18	1.31 (3H, d, 6.6)	15.4
19	1.02 (3H, d, 6.6)	14.0
20	1.21 (3H, d, 6.6)	18.0
1′		175.9
2′	2.67 (1H, m)	33.7
3′/4′	1.14 (3H, d, 7.2)	19.1


### Structure Elucidation

The structures of compounds **1–3** were elucidated by combination of extensive spectroscopic analysis. 1D and 2D NMR spectra were recorded on Bruker Avance III 600 or Agilent DD2-500 spectrometers at 25°C. Chemical shifts were reported with reference to the respective solvent peaks and residual solvent peaks (δ_H_ 2.50 and δ_C_ 39.5 ppm for DMSO-*d*_6_; and δ_H_ 7.26 and δ_C_ 77.1 ppm for CDCl_3_). The molecular weight of each compound was determined using a Q-TOF Ultima Global GAA076 LC-MS spectrometer. CD spectra were recorded on a JASCO J-715 spectropolarimeter, using MeOH as solvent.

### Marfey’s Analysis of Compound **1**

Compound **1** (200 μg) was dissolved in 1 mL of 6 N HCl and hydrolyzed at 110°C for 24 h, and the HCl was then removed by evaporation under the N_2_ gas. The hydrolysate was dissolved in 100 μL of H_2_O and added 200 μL of Marfey’s reagent (1-fluoro-2,4-dinitrophenyl-5-L-alanine amide, FDAA; 1 mg/mL in acetone) together with 50 μL of 1 M NaHCO_3_. Then, the mixture was reacted at 50°C for 60 min. After cooling to room temperature, the mixture was neutralized with 2 N HCl (25 μL). The reaction mixture was subjected to a reversed-phase HPLC (YMC-Pack ODS-A column 150 mm × 4.6 mm, i.d. 5 μm; wavelength: 340 nm) with a linear gradient elution (30–80% solvent B in 50 min; solvent A: H_2_O + 0.1% TFA, solvent B: 90% ACN + 0.1% TFA). Derivatization of L- or D-threonine with FDAA was carried out in the similar manner.

### Mouse Immunization, Cell Culture, and Cytokine (IL-5) Measurement

The inhibitory effects of the compounds **1–3** on the pro-inflammatory cytokine production were determined by the method previously described by Strangman et al. (2009). The experiment was carried out in strict ethical guidelines of Institutional Animal Care and Use Committee. Female BALB/c mice aged 6–8 weeks (Shanghai Laboratory Animal Center, Chinese Academy of Sciences, China), were immunized subcutaneously with ovalbumin (OVA, 25 μg) allergen adsorbed to alum adjuvant (1 mg) in the phosphate-buffered saline (200 μL) weekly for 4 weeks. Then, the mice were sacrificed and their spleens were surgically removed. The splenocytes were separated and suspended in RPMI-1640 media (1.3 × 10^6^ cells/mL) and then were aliquoted into 96 well plates (100 μL/well). Compounds **1–3** (in DMSO) and an aliquot of stimulating OVA allergen were added to the well containing splenocytes, and were incubated for 48 h. The supernatants were then collected and assayed for the cytokine IL-5 production using mouse ELISA kit (R&D Systems Inc.). Absorbance was measured at 450 nm. The wells containing DMSO + OVA allergen were plated as a negative control, and dexamethasone was used as a positive control.

### Cell Viability Assay

Cell viability of human umbilical vein endothelial cells (HUVEC) was measured by MTT assay (Liu G. et al., 2016). The cells seeded into 96-well plate were cultured at 37°C for 24 h, and were treated with various concentrations of SPS (0, 0.2, 0.3, 0.4, 0.5, 0.6, 0.7, 0.8, 0.9, 1.0, and 1.5 mg/mL) for 12 and 24 h. Then MTT solution (5 mg/mL, 20 μL) was added to each well, and were incubated for 4 h. After that, the medium was removed and DMSO (150 μL) was added to each well to dissolve purple crystals of formazan at 260 rpm for 10 min. Absorbance was measured with TECAN infinite M1000 Pro multi-detection microplate reader at 490 nm and relative cell viability was presented as a percentage relative to the control group. The LD_50_ value was determined as the concentration that caused 50% inhibition of cell proliferation.

### Antibacterial Activity Assay

The antibacterial activity against five multi-drug resistant (MDR) strains (*S. aureus* CCARM 3090, *Escherichia coli* CCARM 1009, *Enterococcus faecalis* CCARM 5172, *Enterococcus faecium* CCARM 5203, *Salmonella typhimurium* CCARM 8250) was tested by the radial diffusion assay. Each bacterial strain was grown overnight at 37°C in LB media and diluted to 1/100. A gel solution containing 2.5% (w/v) of powdered LB medium and 1.5% agar was prepared and autoclaved. Then, 0.15 mL of the diluted bacterial culture was added to 15 mL of the gel solution at 40–50°C. Once the bacteria were adequately dispersed, the gel was poured into a plate (90 mm × 15 mm). After solidification, wells were made using a 2 mm punch. Each sample (30 mg/mL, 10 μL) was added to the well, and the plates were incubated for 18 h at 37°C. Tetracycline (≥98%, 30 μg/well) was used as a positive control. The diameters of the inhibition zones surrounding the wells were measured in millimeters.

## Results

### Isolation and Purification of the Compounds

The Δ*wblA_so_* mutant strain of *S. somaliensis* SCSIO ZH66 was obtained as described in our previous study (Huang et al., 2016). The fermentation broths of the wild-type and the Δ*wblA_so_* mutant strains were extracted with EtOAc, respectively, and were subsequently subjected to HPLC analysis, in which we observed a series of peak changes (**Figure 2**). The enhanced peaks of **a** and **b** in Δ*wblA_so_* strain displayed similar UV spectra with characteristic UV absorption around 230 and 320 nm, indicating them likely to be the same class of compounds. With the large scale fermentation of the Δ*wblA_so_* mutant strain and further UV-spectrum-guided separation, we isolated a major compound **1** together with a small amount of compound **2** from peak **b**, and compound **3** from peak **a**.

### Structural Identification of Compounds **1–3**

**FIGURE 3:**
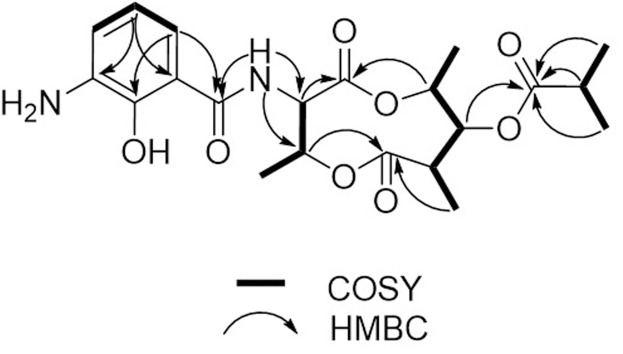
**^1^H-^1^H COSY and key HMBC correlations of **1****.

Compound **1** was isolated as a brown, amorphous solid. The molecular formula of **1** was established as C_21_H_28_O_8_N_2_ on the basis of HR-ESIMS data ([M + H]^+^ at *m/z* 437.1931; Supplementary Figure S4). The planar structure of **1** was determined by 1D (^1^H, ^13^C) and 2D NMR (COSY, HSQC, and HMBC) data (Supplementary Figures S5–S10). The structure elucidation step was first started with a secondary amide proton NH-10 (δ_H_ 9.26), which acts as a connection bridge between two parts of substructures. In the ^1^H-^1^H COSY spectrum, we observed two proton spin systems that consist of NH-10/H-3 (δ_H_ 5.28)/H-4 (δ_H_ 5.54)/H-18 (δ_H_ 1.31), and H-19 (δ_H_ 1.02)/H-7 (δ_H_ 2.65)/H-8 (δ_H_ 4.83)/H-9 (δ_H_ 4.92)/H-20 (δ_H_ 1.21), respectively (**Figure 3**). The ^13^C chemical shifts of C-2 (δ_C_ 169.9), C-4 (δ_C_ 71.8), C-6 (δ_C_ 174.5), and C-9 (δ_C_ 73.9), as well as the ^1^H chemical shifts of H-4 and H-9 revealed the existence of two ester groups. Additionally, the HMBC correlations (**Figure 3**) from H-3 and H-9 to C-2, and from H-4, H-7, and H-8 to C-6 finally assigned the substructure as a nine-membered *bis*-lactone ring. The HMBC correlations from NH-10 and H-17 (δ_H_ 7.74) to the carbonyl carbon C-11 (δ_C_ 170.2) allowed further assignment of the substructure located on the other side of the amide group. The ^1^H-^1^H COSY correlations between H-15 (δ_H_ 7.20), H-16 (δ_H_ 6.87) and H-17, together with the HMBC correlations from these protons to three quaternary carbons C-12 (δ_C_ 115.0), C-13 (δ_C_ 151.4), and C-14 (δ_C_ 129.1) revealed that a trisubstituted aromatic ring connected to the amide group. The downfielded ^13^C chemical shift value of C-13 compared to other aromatic carbons proved it to be a hydroxylated carbon. The HMBC correlations from H-8, H-2′ (δ_H_ 2.67), H-3′/H-4′ (δ_H_ 1.14) to C-1′ (δ_C_ 175.9) revealed an isobutyrate moiety was attached to C-8 of the *bis*-lactone ring. Based on the HR-ESIMS data of **1**, we finally confirmed that C-14 was an aminated aromatic carbon, thus assigning the planar structure of **1** as an antimycin-type depsipeptide.

**FIGURE 4:**
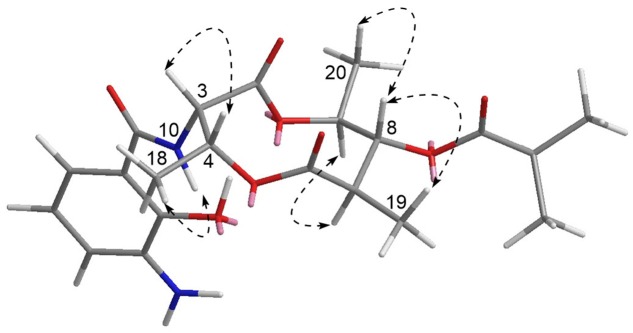
**Key NOESY correlations of **1**.** The 3D structure is the conformer with the lowest energy.

Compound **1** has five chiral carbons (C-3, C-4, C-7, C-8, and C-9), all of which are located on the *bis*-lactone ring. The strong NOE correlations of H-18/NH-10 and H-3/H-4 (**Figure 4**), as well as small *J* value (7.2 Hz) between H-3 and H-4 provided credible evidence for the assignment of *syn* configuration between C-3 and C-4. Furthermore, C-3 and C-4 are part of the threonine residue (C^α^ and C^β^, respectively) embedded in **1**. Therefore, we performed acid-catalyzed hydrolysis followed by Marfey’s analysis to determine their absolute configurations (Supplementary Figure S1). According to the result, the threonine residue in **1** was identified to be L-amino acid; hence the absolute configurations of C-3 and C-4 were assigned to be *S*- and *R*-, respectively. In the ^1^H NMR spectrum, H-8 showed a triplet signal pattern with the *J* value of 10.2 Hz, indicative of the *anti*-configurations between C-7, C-8, and C-9. The NOE correlations of H-7/H-9, H-8/H-19 and H-8/H-20 further supported this assignment (**Figure 4**). In our attempt to determine the absolute configurations of C-7, C-8, and C-9 using ECD calculation, it was failed because of the high structural flexibility. Nevertheless, the ^1^H and ^13^C chemical shifts of *bis*-lactone ring in **1** showed almost identical values with those of the reported antimycin-type depsipeptide USF-19A (**2**) (Komoda et al., 1995), in which the 3-aminosalicylate of **1** is replaced by a 3-formamidosalicylate moiety. The chemical shifts similarity of C-7, C-8, and C-9 in two compounds indicated that these chiral carbons in **1** share the same absolute configurations with those in USF-19A, which were determined as *7R*, *8R*, and *9S*, respectively. The ^1^H and ^13^C NMR chemical shift values of **1** are summarized in **Table 1**.

Compound **2** was isolated as a white, amorphous solid. The molecular formula of **2** was established as C_22_H_28_O_9_N_2_ on the basis of HR-ESIMS data ([M + H]^+^ at *m/z* 465.1869) (Supplementary Figure S11). Analysis of its NMR data recorded in DMSO-*d*_6_ (Supplementary Figures S12–S19 and Table S1) revealed that its structure resembles that of **1** except for the amide linkage of a formyl group at C-14 (δ_C_ 127.4), which was determined by the HMBC correlations from H-22 (δ_H_ 8.34) to C-14 and from NH-21 (δ_H_ 9.85) to C-22 (δ_C_ 160.8) (Supplementary Figure S15). Thus, **2** was identified as USF-19A, that was further collaborated by comparison of ^1^H and ^13^C NMR data (in CDCl_3_) with those reported (Komoda et al., 1995).

Compound **3** was isolated as a white, amorphous solid. The molecular formula of **3** was established as C_18_H_22_O_8_N_2_ on the basis of HR-ESIMS data ([M + H]^+^ at *m/z* 395.1441) (Supplementary Figure S20). Compound **3** has a free hydroxyl group at C-8 (δ_C_ 77.2) instead of an isobutyrate moiety in **1** and **2**. Compound **3** was identified as urauchimycin D by NMR assignment with further comparison of ^1^H and ^13^C NMR data (Supplementary Figures S21–S25 and Table S1) with those reported (Yao et al., 2006).

### Inhibitory Activity on IL-5 Production

**FIGURE 5:**
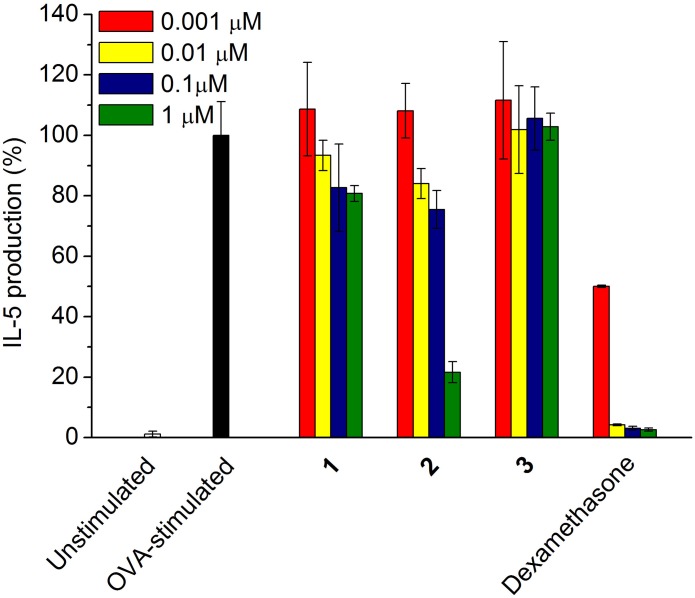
**Inhibitory effect of compounds **1–3** on the pro-inflammatory cytokine IL-5 production.** OVA-stimulated splenocytes were incubated with each sample from 0.001 to 1.0 μM, respectively. Dexamethasone was used as a positive control.

To investigate the potent anti-inflammatory activity of the compounds **1–3**, the inhibitory effect of each compound on the cytokine (IL-5) production was tested dose dependently. According to **Figure 5**, compound **2** exhibited up to 80% of inhibition on IL-5 production in the OVA-stimulated splenocytes at the concentration of 1 μM, the IC_50_ value of which was further determined as 0.57 μM; however, **1** and **3** showed only less than 20% and null activity, respectively, in the concentration ranging from 0.001 to 1 μM.

### Cytotoxicity Assay

To evaluate the cytotoxicity of compounds **1–3** toward human normal cells, the cell viability assay was performed, in which compounds **1–3** showed very weak cytotoxicity toward HUVEC with LD_50_ values of 62.6, 34.6, and 192.9 μM, respectively (Supplementary Table S2).

### Antibacterial Activity Assay

In the antibacterial activity evaluation, compounds **1–3** exhibited null inhibition zones against five MDR strains (*S. aureus* CCARM 3090, *E. coli* CCARM 1009, *E. faecalis* CCARM 5172, *E. faecium* CCARM 5203, *S. typhimurium* CCARM 8250) at the treating amount of 300 μg/well (data not shown).

## Discussion

Antimycin-type compounds are a class of depsipeptides sharing a nine-membered *bis*-lactone ring with a conserved substitution of 3-formamidosalicylic acid, which exhibited a wide range of bioactivities including antifungal, insecticidal, antiviral, anticancer, and anti-inflammatory (Hosotani et al., 2005; Shiomi et al., 2005; Raveh et al., 2013; Liu J. et al., 2016). Antimycins are generated from a hybrid NRPS (non-ribosomal peptide synthetase)–PKS (polyketide synthase) assembly line in various *Streptomyces* species with the 3-formamidosalicylate substitution as a starter unit.

In the present study, we isolated an unusual 3-aminosalicylate substituted somalimycin (**1**) from the Δ*wblA_so_* mutant strain of *S. somaliensis* SCSIO ZH66, which was accumulated after the disruption of the negative global regulatory gene *wblA_so_*. Recently, Zhang and colleagues reconstituted the 3-formamidosalicylate moiety in *E. coli*, revealing that it is generated from anthranilic acid by the action of the multicomponent oxygenase AntHIJKL and the formyltransferase AntO on the carrier protein AntG. The intermediates during this process, including anthraniloyl-*S*-AntG and 3-aminosalicyloyl-*S*-AntG, could be recognized by the following hybrid NRPS–PKS assembly line to yield shunt products with anthranilate and 3-aminosalicylate substitution, respectively (Liu et al., 2015). The significantly increased production of 3-aminosalicylate substituted somalimycin (**1**) might be the result of the accumulation of 3-aminosalicyloyl-*S*-AntG, which could be explained by the up-regulation of AntHIJKL in the *wblA_so_* mutant.

In our investigation for potent anti-inflammatory activity of the compounds **1–3**, USF-19A (**2**) exhibited highest inhibition on IL-5 production in the OVA-stimulated splenocytes (IC_50_ = 0.57 μM); however, somalimycin (**1**) and urauchimycin D (**3**) showed only less than 20% and null activity, respectively, up to the concentration of 1 μM (**Figure 5**). Fenical and colleagues reported that splenocins A–J, a subfamily of antimycin-type depsipeptides, exhibited strong inhibition against IL-5 production, some of which are comparable to that of the corticosteroid drug dexamethasone (Strangman et al., 2009). Among them, splenocin B exhibited the strongest inhibition with IC_50_ value of 1.8 nM, while splenocin J showed the weakest activity (IC_50_ = 1022.7 nM). The activity gap between splenocins B and J is over 500-fold, and somalimycin (**1**) and urauchimycin D (**3**) showed even much lower activity (IC_50_ > 10 μM) than splenocin J. From a structural point of view, compounds **1–3** and splenocins share a common structural skeleton: a nine-membered *bis*-lactone ring with an amide linkage (C-3) connecting to a salicylic acid. The structural differences among these compounds are substitutions at C-7, C-8, and C-14. By comparable analysis of the bioactivity results in this study with those of splenocins (Strangman et al., 2009), we came to a conclusion that (i) the ester linkage to the hydroxyl group at C-8 plays the most important role in the inhibition of IL-5 production; (ii) and the longer alkyl group at C-7 displays the stronger activity; (iii) furthermore, the formylation of the amine group at C-14 is also essential for enhancement of IL-5 inhibition. Compounds **1–3** showed very weak cytotoxicity against HUVEC (Supplementary Table S2), which were far over their IL-5 inhibitory activity. Thus, these compounds may have good potential for further use in the development of anti-inflammatory drugs. Furthermore, the SAR study of the antimycin-type depsipeptides on the IL-5 production in this study may provide useful information for searching or synthesizing novel antimycin-based anti-inflammatory lead compounds.

## Conclusion

We isolated a new (**1**) and two known (**2** and **3**) antimycin-type depsipeptides from the Δ*wblA_so_* mutant strain of deepsea-derived *S. somaliensis* SCSIO ZH66. USF-19A (**2**) exhibited strong inhibition against IL-5 production (IC_50_ = 0.57 μM), while somalimycin (**1**) and urauchimycin D (**3**) showed much weaker activity (IC_50_ > 10 μM), which might be attributed to their different side-chain substitutions at C-8 and C-14. All of these three compounds showed very low cytotoxicity against HUVEC, which provided the possibility for their further use in anti-inflammatory drug development.

## Ethics Statement

This study was carried out in accordance with the recommendations of the Animal Research and Ethics Committee of Ocean University of China. The protocol was approved by the Animal Research and Ethics Committee of Ocean University of China.

## Author Contributions

HL was involved in the NMR assignment and wrote the manuscript. HH and LH performed experiments. JJ provided *S. somaliensis* SCSIO ZH66. WL supervised the whole work and revised and edited the manuscript. All authors read and approved the final manuscript.

## Conflict of Interest Statement

The authors declare that the research was conducted in the absence of any commercial or financial relationships that could be construed as a potential conflict of interest.
