# Supplementary material for: Discovery of Antimycin-Type Depsipeptides from a wbl Gene Mutant Strain of Deepsea-Derived Streptomyces somaliensis SCSIO ZH66 and Their Effects on Pro-inflammatory Cytokine Production
Source: Front Microbiol. 2017 Apr 19;8:678. doi: 10.3389/fmicb.2017.00678 (PMC5395633; doi:10.3389/fmicb.2017.00678)
Supplement: Supplementary file 1 [file Image_1.pdf]

Supplementary Material

Discovery of Antimycin-Type Depsipeptides from a *wbl* Gene Mutant Strain of Deepsea-Derived *Streptomyces somaliensis* SCSIO ZH66 and their effects on Pro-Inflammatory Cytokine Production

Huayue Li, Huiming Huang, Lukuan Hou, Jianhua Ju, Wenli Li*

*** Correspondence:** Wenli Li: liwenli@ouc.edu.cn

Table of Contents

Figure S1. HPLC chromatograms of FDAA derivatives of standard threonine and acid hydrolysate of **1**

Figure S2. UV-spectrum of **1**

Figure S3. CD spectrum of **1**

Figure S4. The HR-ESIMS spectrum of **1**

Figure S5. ^{1}H NMR spectrum of **1** in DMSO-d_6

Figure S6. ^{13}C NMR spectrum of **1** in DMSO-d_6

Figure S7. COSY spectrum of **1** in DMSO-d_6

Figure S8. HSQC spectrum of **1** in DMSO-d_6

Figure S9. HMBC spectrum of **1** in DMSO-d_6

Figure S10. NOESY spectrum of **1** in DMSO-d_6

Figure S11. The HR-ESIMS spectrum of **2**

Figure S12. ^{1}H NMR spectrum of **2** in DMSO-d_6

Figure S13. COSY spectrum of **2** in DMSO-d_6

Figure S14. HSQC spectrum of **2** in DMSO-d_6

Figure S15. HMBC spectrum of **2** in DMSO-d_6

Figure S16. ^{1}H NMR spectrum of **2** in $CDCl_3$

Figure S17. COSY spectrum of **2** in $CDCl_3$

Figure S18. HSQC spectrum of **2** in $CDCl_3$

Figure S19. HMBC spectrum of **2** in $CDCl_3$

Figure S20. The HR-ESIMS spectrum of **3**

Figure S21. ^{1}H NMR spectrum of **3** in DMSO-d_6

Figure S22. ^{13}C NMR spectrum of **3** in DMSO-d_6

Figure S23. COSY spectrum of **3** in DMSO-d_6

Figure S24. HSQC spectrum of **3** in DMSO-d_6

Figure S25. HMBC spectrum of **3** in DMSO-d_6

Table S1. ^{1}H and ^{13}C NMR chemical shifts of **2** and **3** in DMSO-d_6

Table S2. Cytotoxicity of compounds **1**−3 against human umbilical vein endothelial cells (HUVEC).

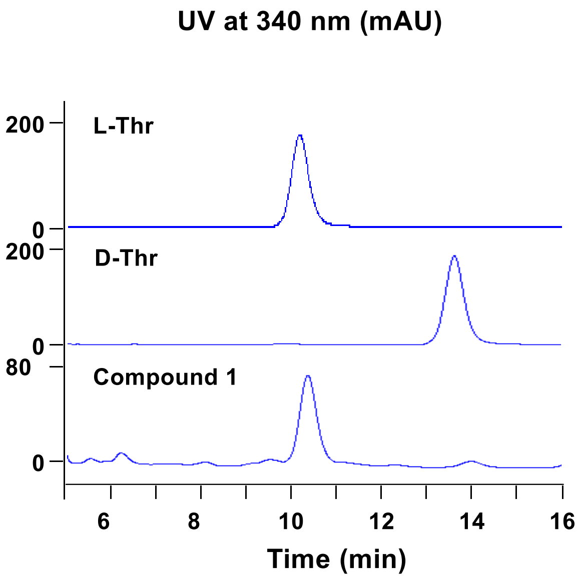

Figure S1. HPLC chromatograms of FDAA derivatives of standard threonine and acid hydrolysate of compound **1**

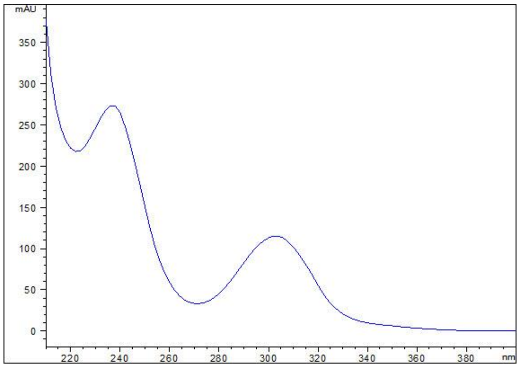

Figure S2. UV-spectrum of **1**

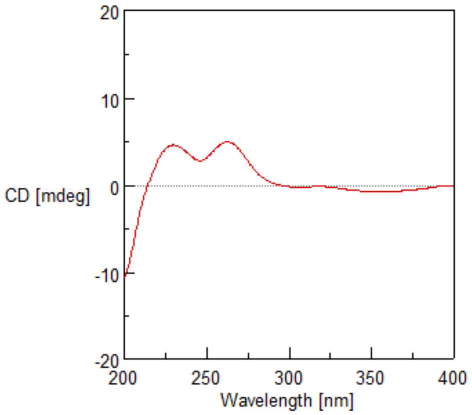

Figure S3. CD spectrum of **1**

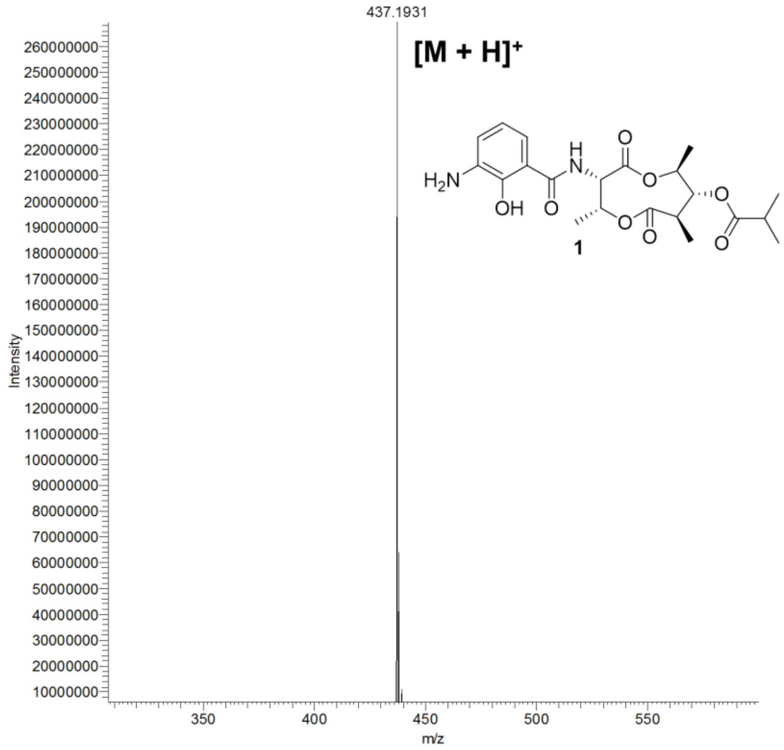

Figure S4. The HR-ESIMS spectrum of **1**

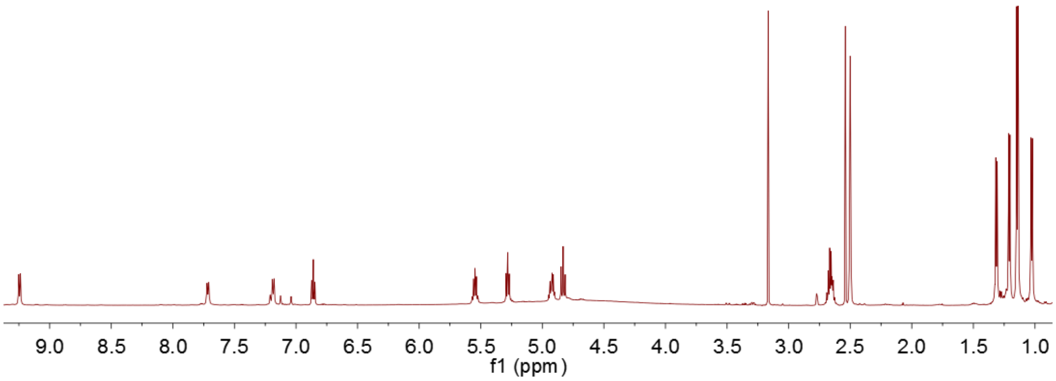

Figure S5. ^{1}H NMR spectrum of **1** in DMSO-d_6

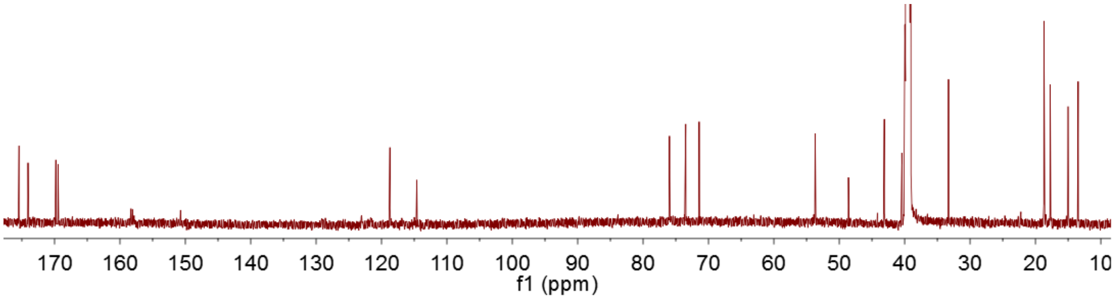

Figure S6. ^{13}C NMR spectrum of **1** in DMSO-d_6

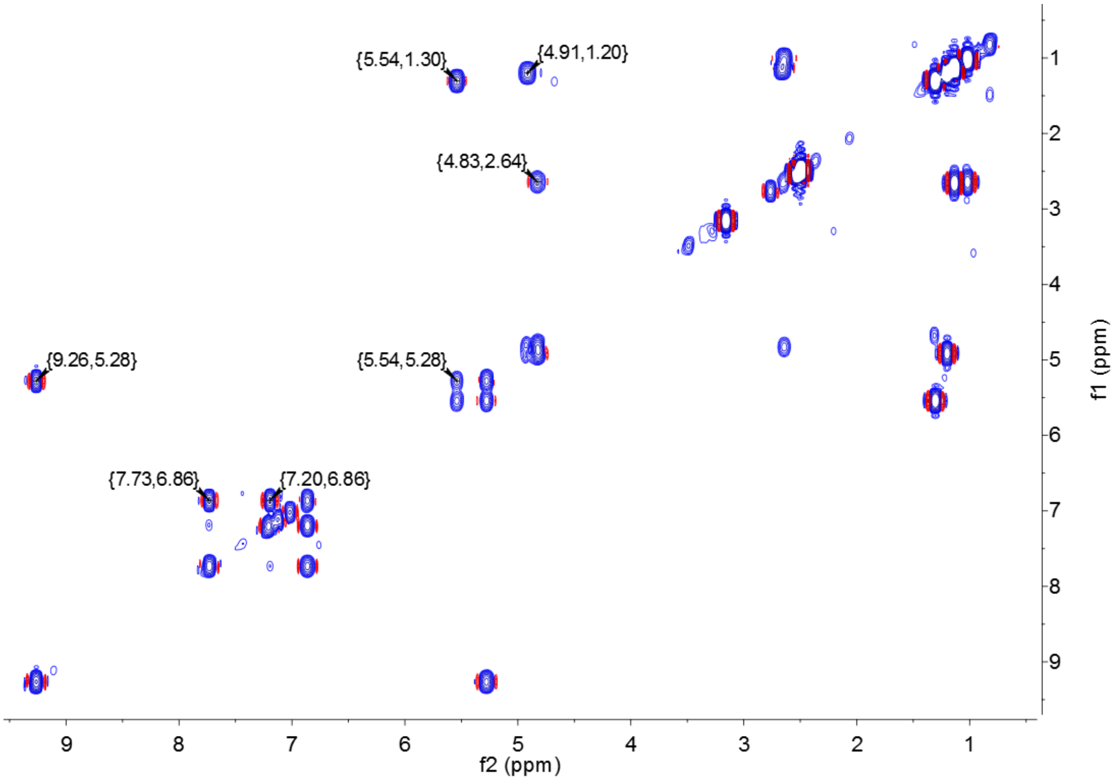

Figure S7. COSY spectrum of **1** in DMSO-d_6

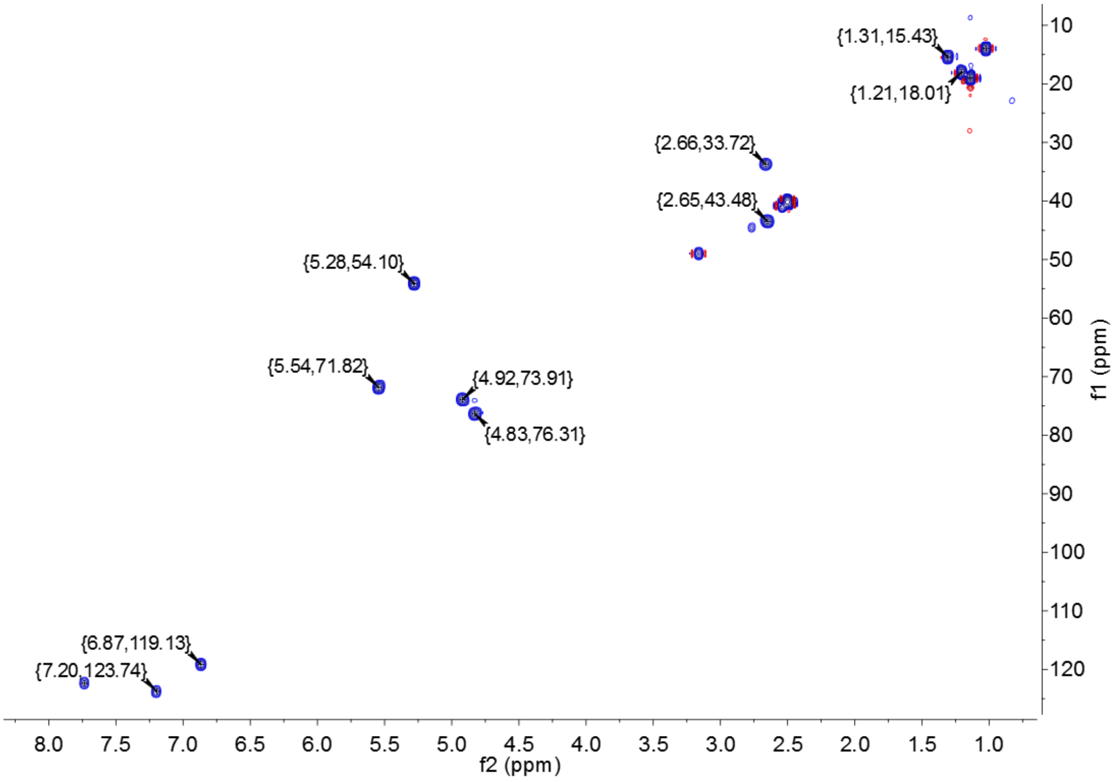

Figure S8. HSQC spectrum of **1** in DMSO-d_6

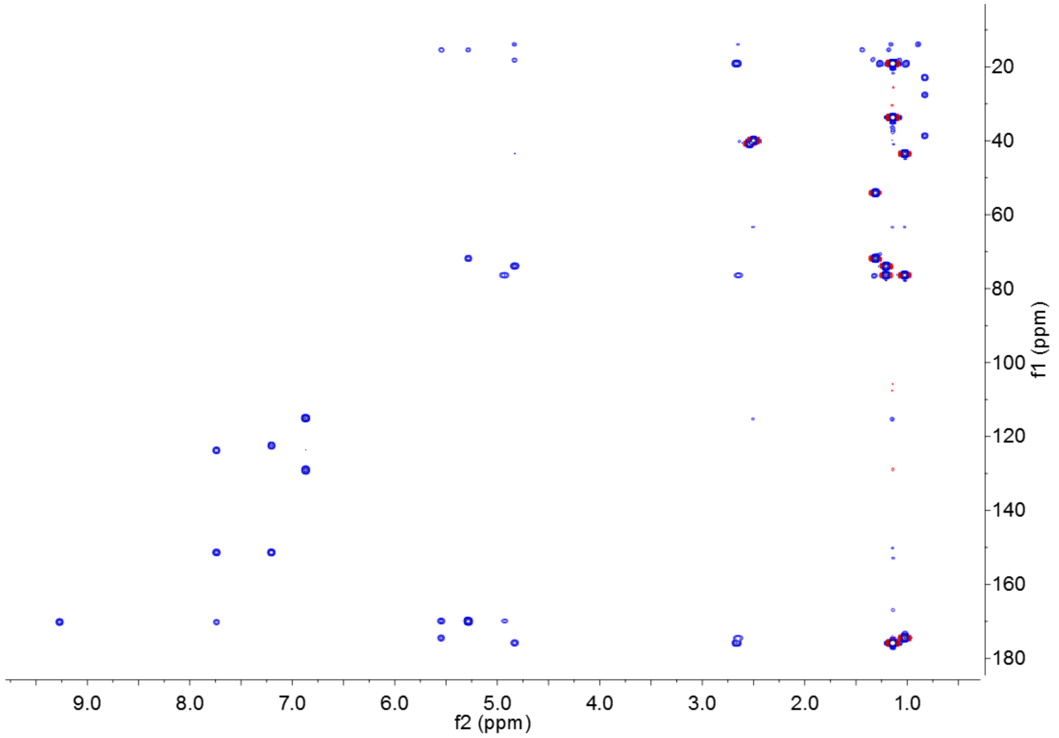

Figure S9. HMBC spectrum of **1** in DMSO-d_6

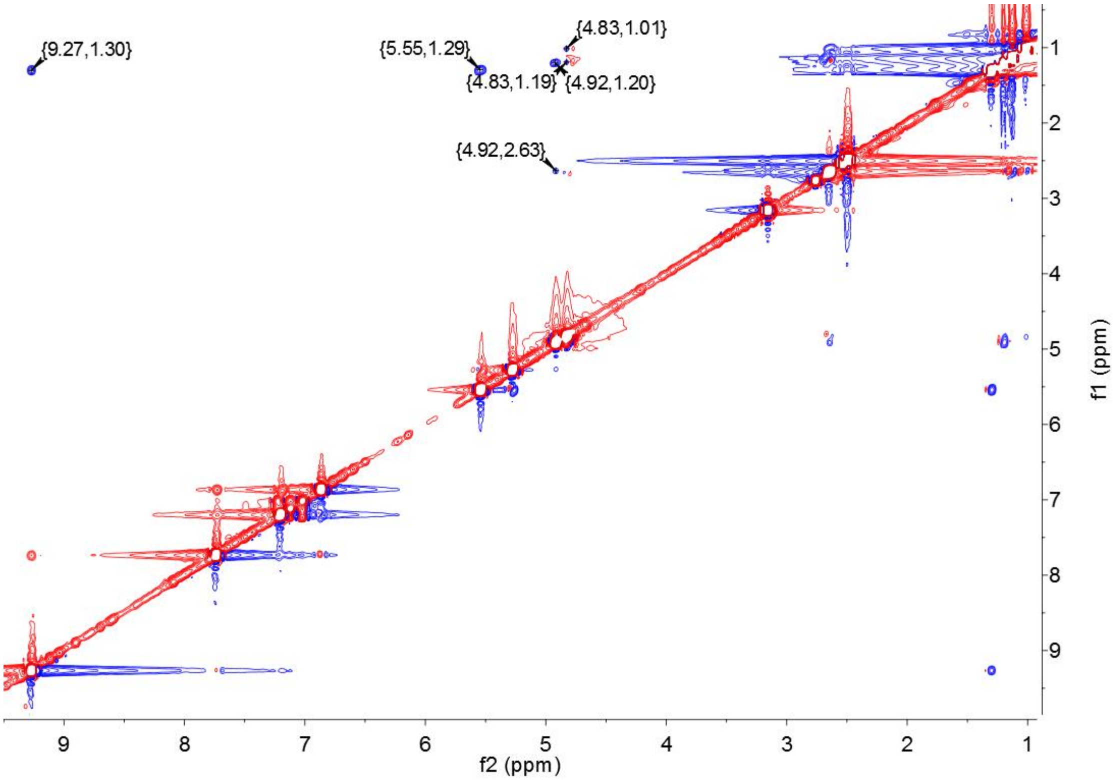

Figure S10. NOESY spectrum of **1** in DMSO-d_6

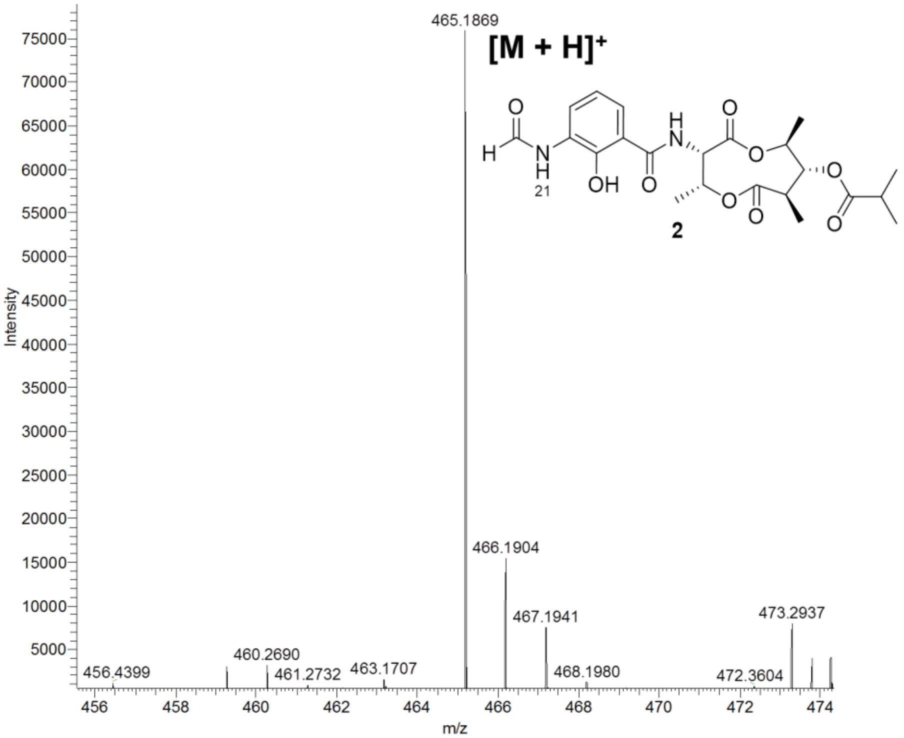

Figure S11. The HR-ESIMS spectrum of **2**

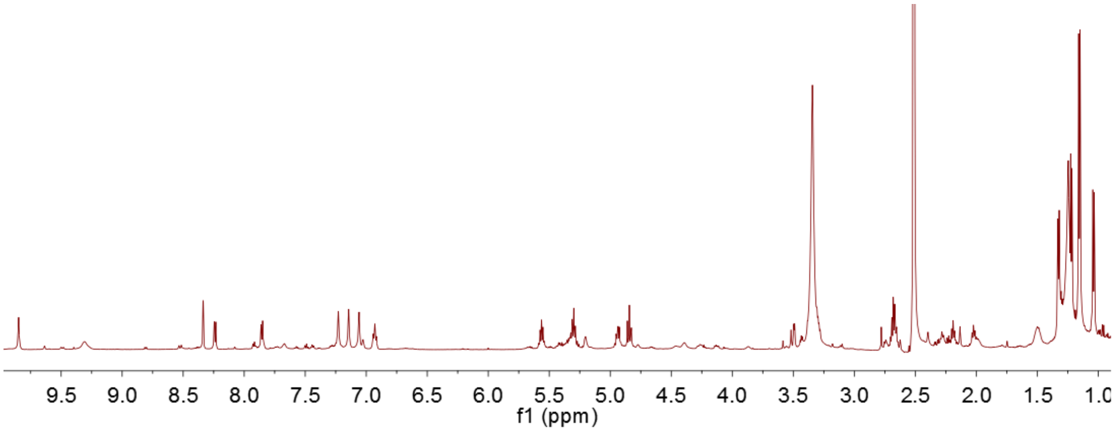

Figure S12. ^{1}H NMR spectrum of **2** in DMSO-d_6

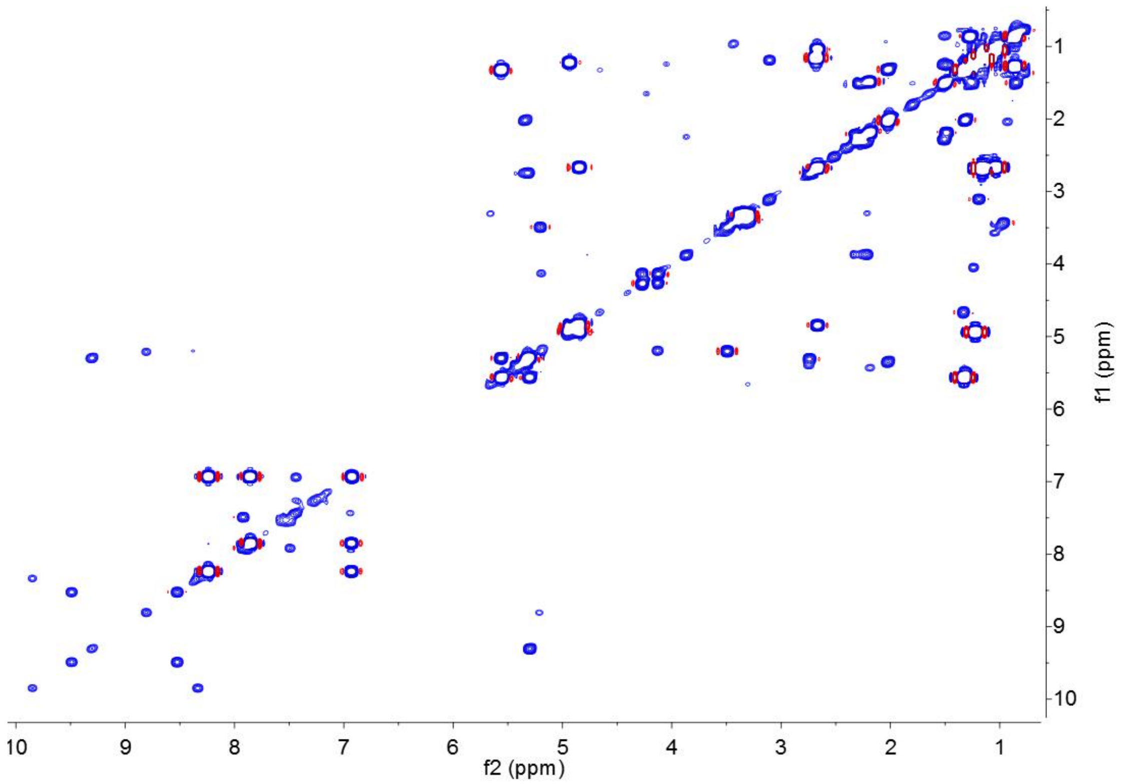

Figure S13. COSY spectrum of **2** in DMSO-d_6

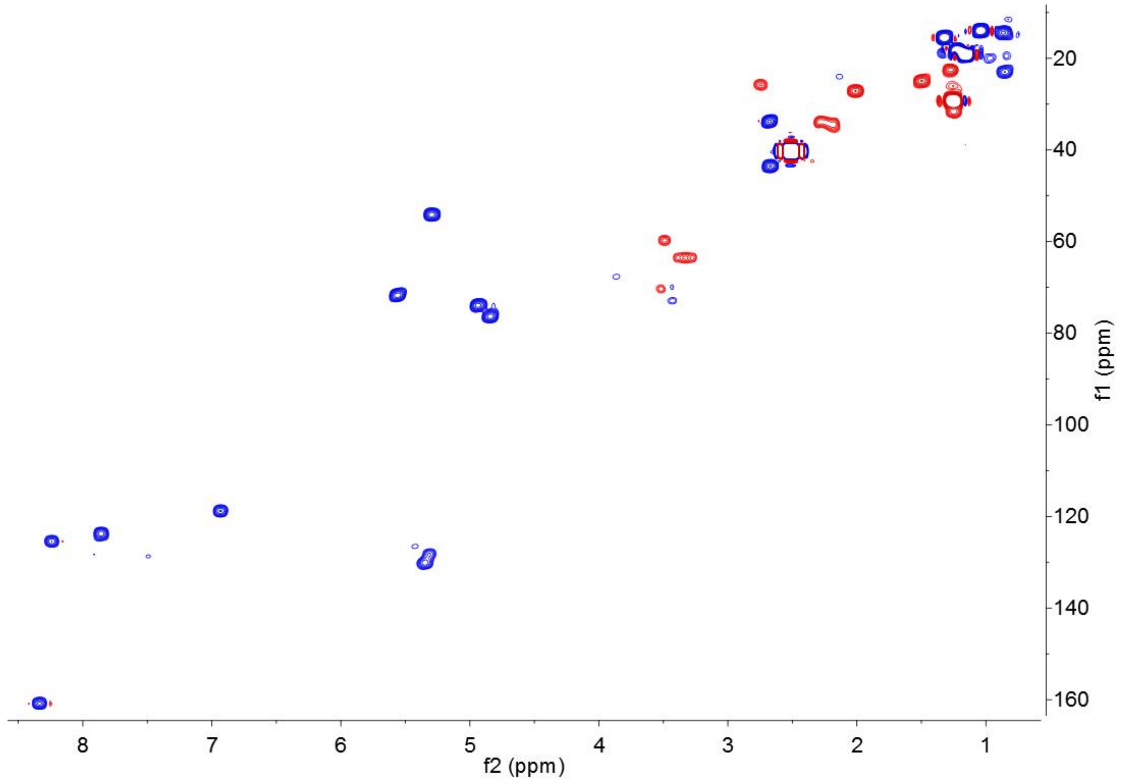

Figure S14. HSQC spectrum of **2** in DMSO-d_6

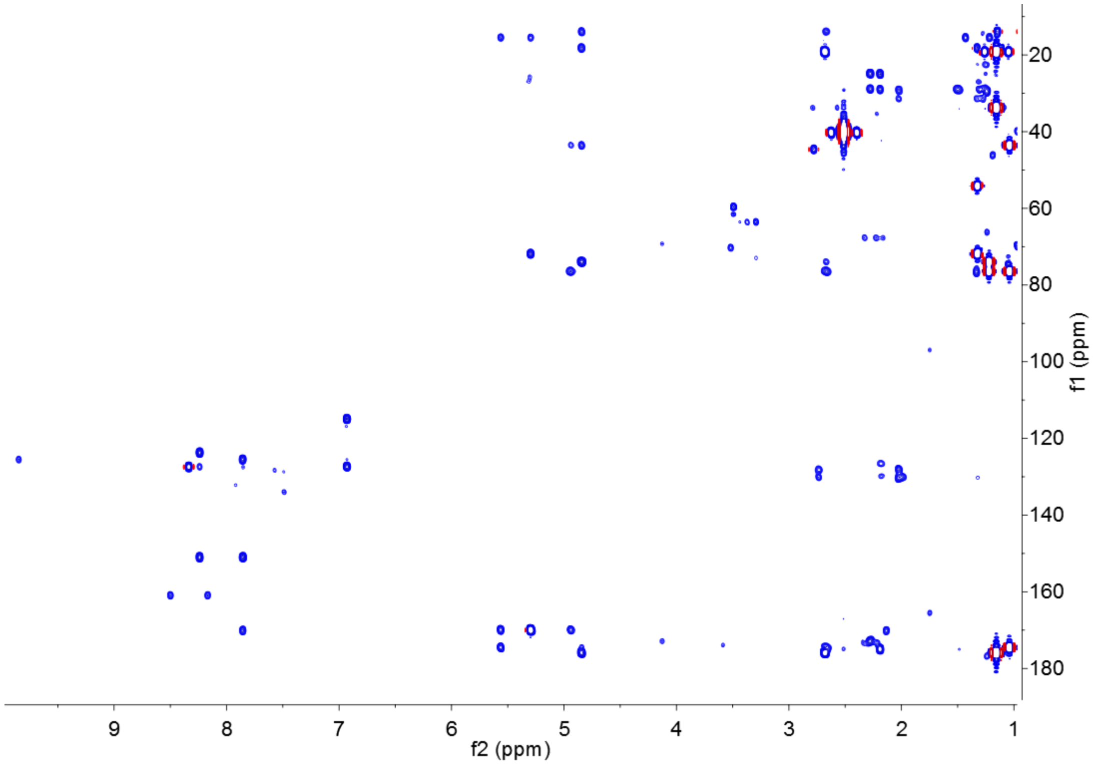

Figure S15. HMBC spectrum of **2** in DMSO-d_6

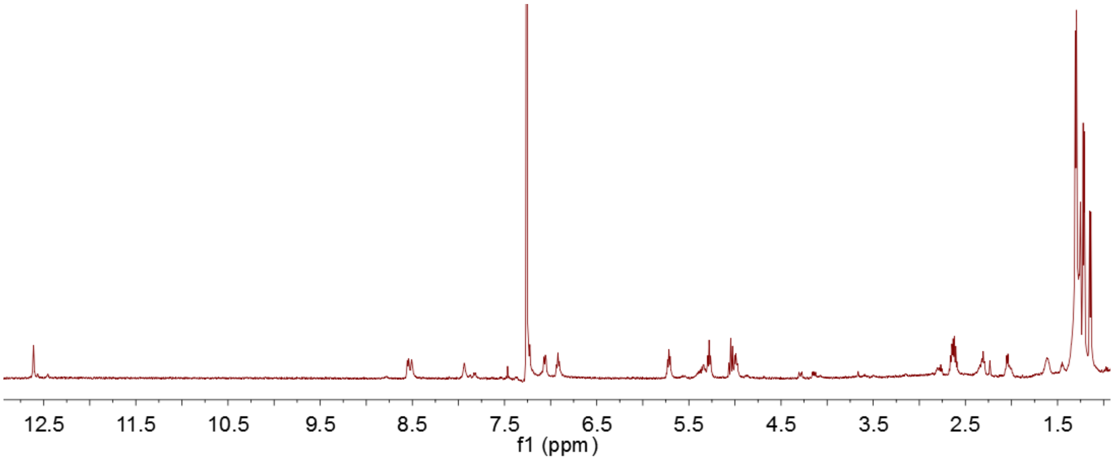

Figure S16. ^{1}H NMR spectrum of **2** in $CDCl_3$

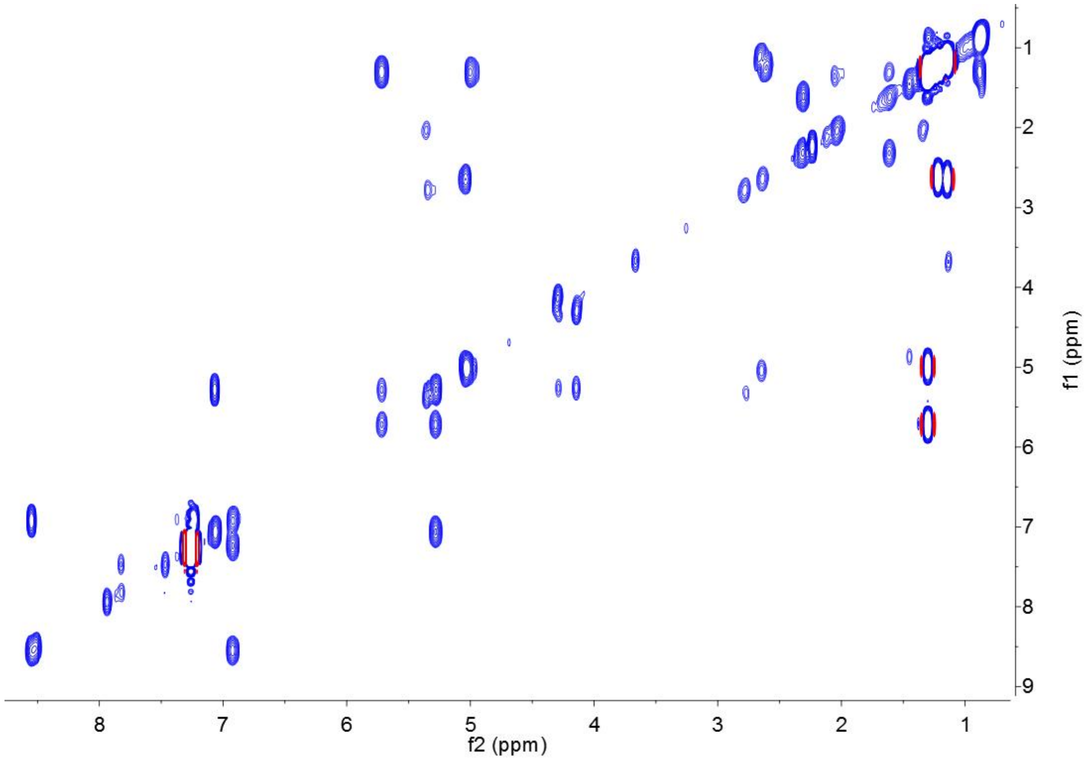

Figure S17. COSY spectrum of **2** in $CDCl_3$

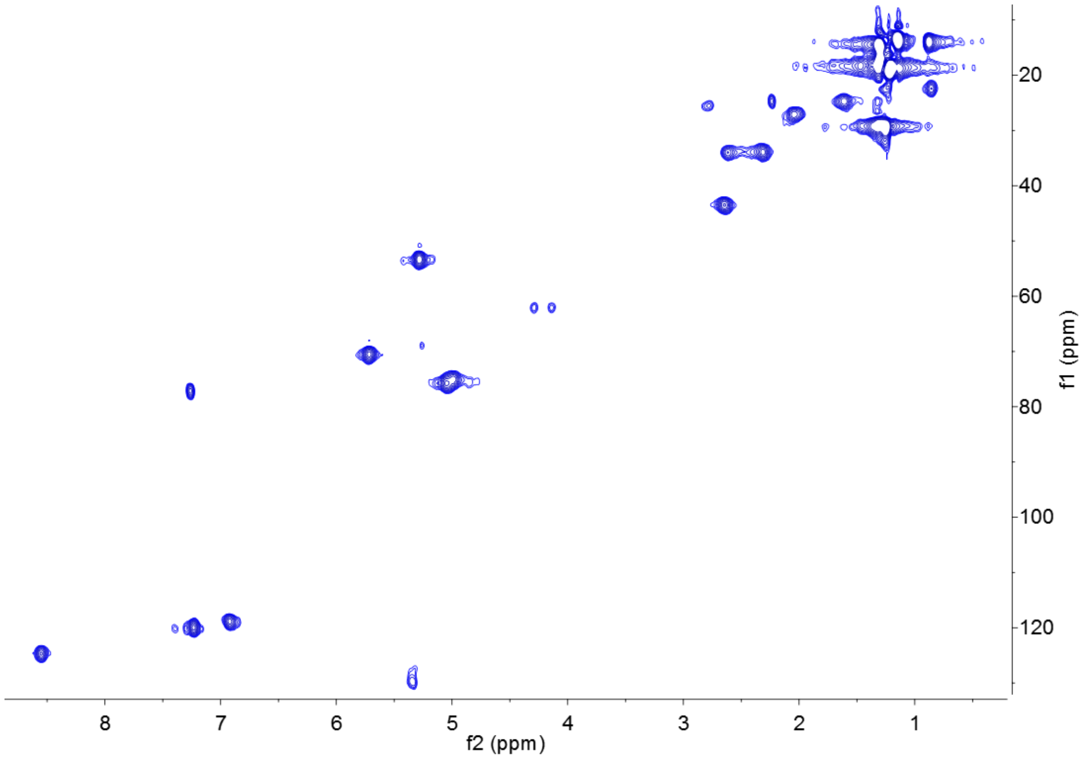

Figure S18. HSQC spectrum of **2** in $CDCl_3$

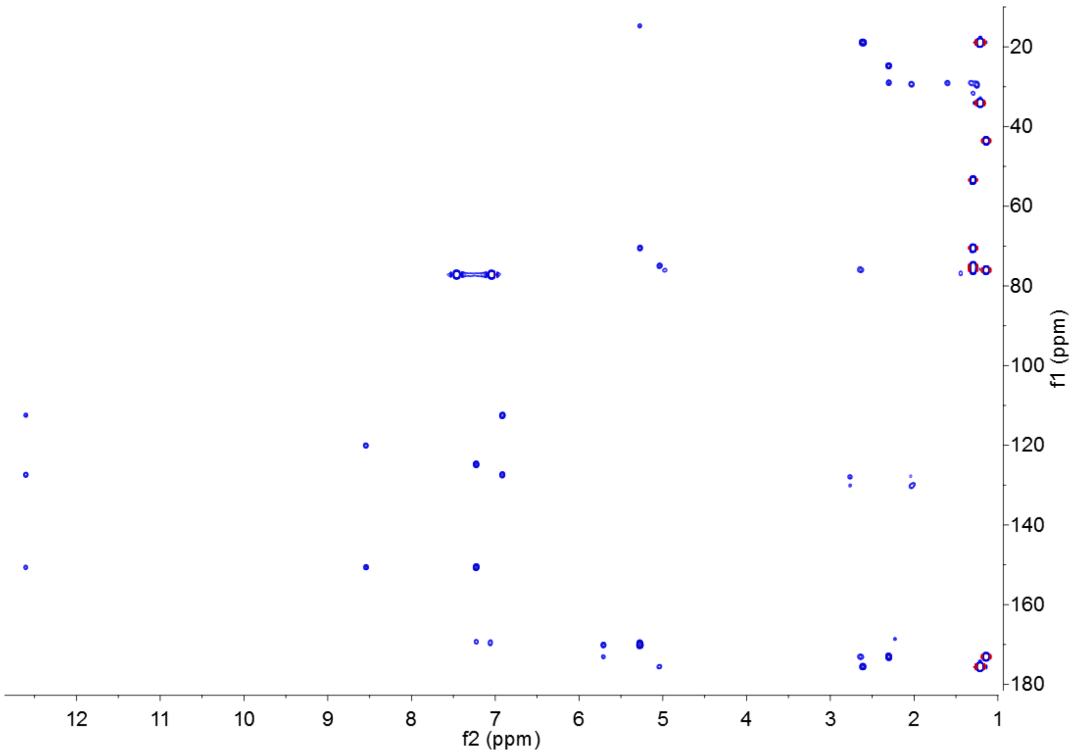

Figure S19. HMBC spectrum of **2** in $CDCl_3$

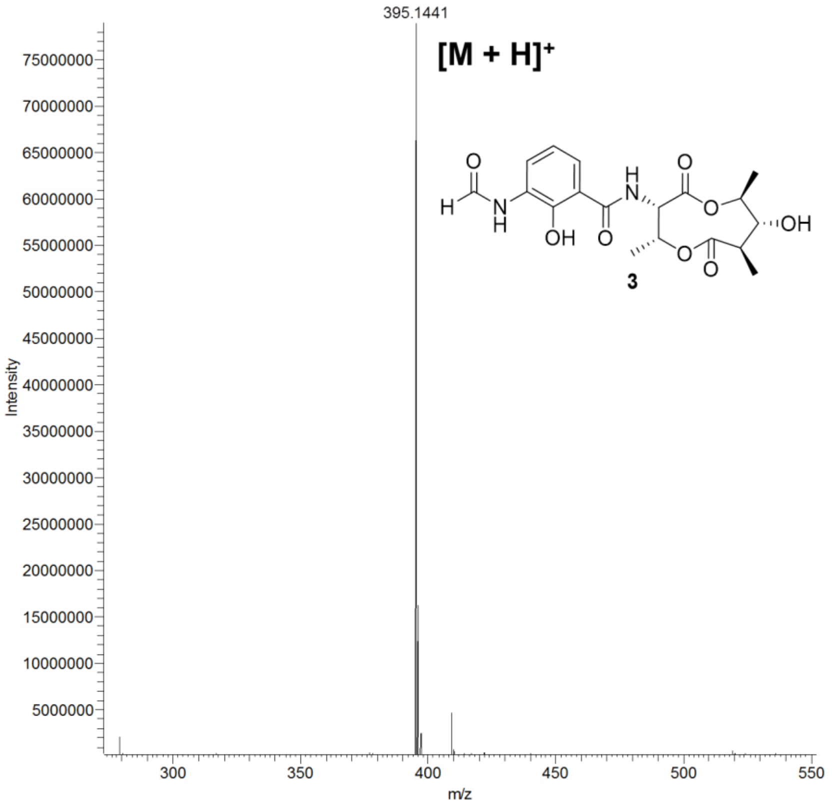

Figure S20. The HR-ESIMS spectrum of **3**

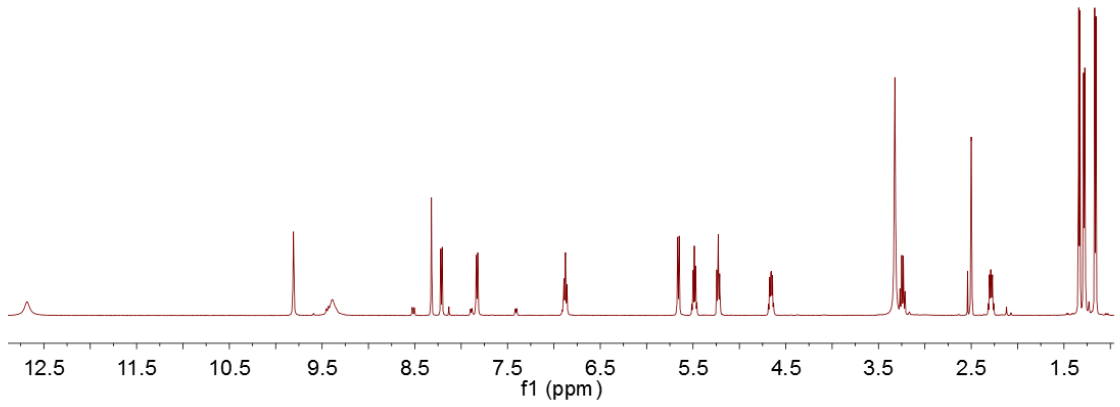

Figure S21. ^{1}H NMR spectrum of **3** in DMSO-d_6

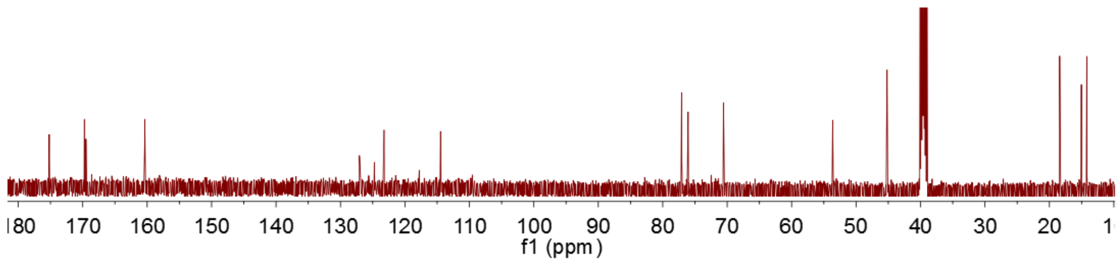

Figure S22. ^{13}C NMR spectrum of **3** in DMSO-d_6

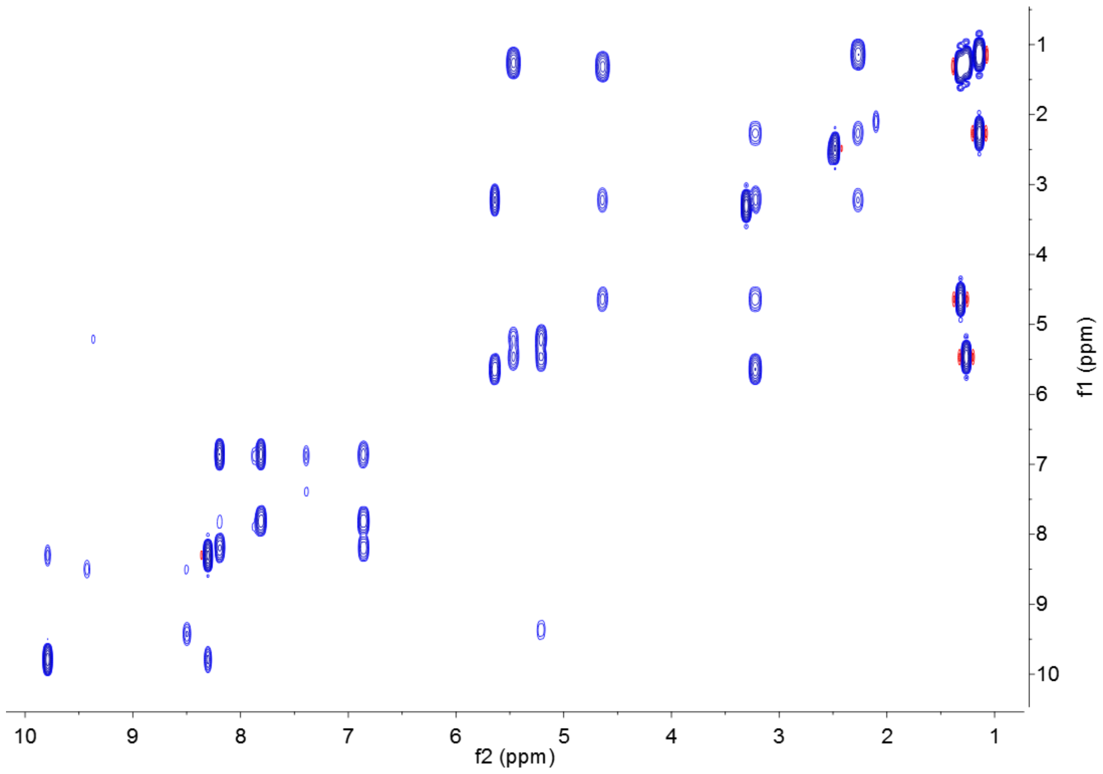

Figure S23. COSY spectrum of **3** in DMSO-d_6

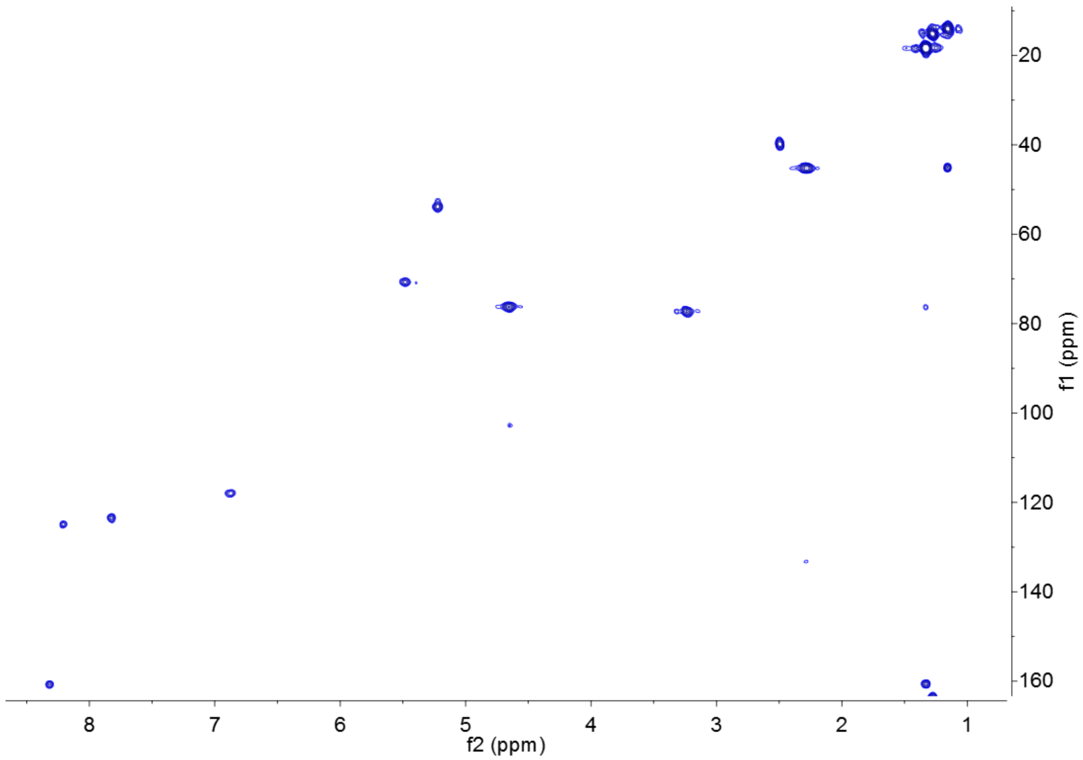

Figure S24. HSQC spectrum of **3** in DMSO-d_6

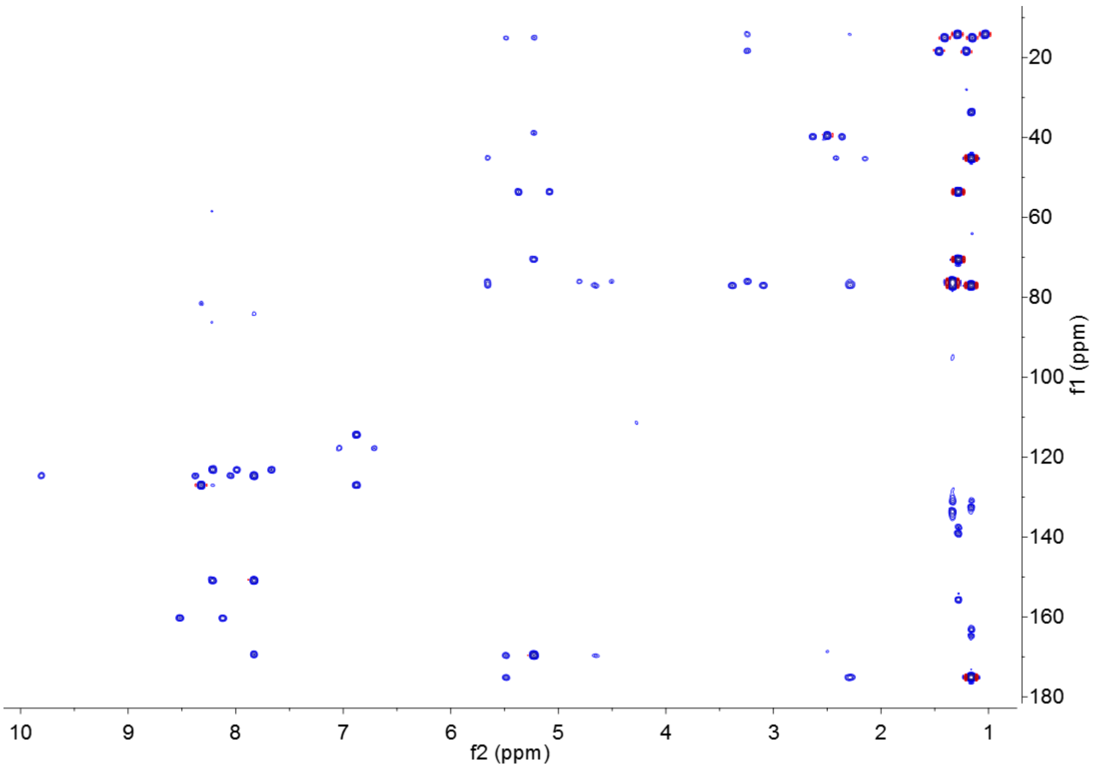

Figure S25. HMBC spectrum of **3** in DMSO-d_6

Table S1. ^{1}H and ^{13}C NMR chemical shifts of **2** and **3** in DMSO-d_6

	**2**		**3**	
Position	δ_H (J in Hz)	δ_C	δ_H (J in Hz)	δ_C
2		169.9		169.7
3	5.30 (1H, t, 7.8)	54.1	5.22 (1H, t, 7.5)	53.8
4	5.56 (1H, m)	71.7	5.48 (1H, quin, 7.0)	70.7
6		174.5		175.2
7	2.67 (1H, m)	43.5	2.28 (1H, m)	45.2
8	4.84 (1H, t, 10.2)	76.3	3.23 (1H, quar, 9.0)	77.2
9	4.94 (1H, m)	73.9	4.65 (1H, m)	76.2
10-NH	9.31 (1H, bs)		9.36 (1H, bs)	
11		170.1		169.5
12		114.9		114.5
13		151.0		150.9
14		127.4		127.0
15	8.24 (1H, d, 7.8)	125.4	8.21 (1H, d, 7.5)	124.8
16	6.93 (1H, t, 7.8)	118.8	6.87 (1H, t, 7.5)	117.9
17	7.86 (1H, d, 7.8)	123.8	7.83 (1H, d, 7.5)	123.4
18	1.32 (3H, d, 6.6)	15.5	1.27 (3H, d, 6.5)	15.2
19	1.04 (3H, d, 6.6)	13.9	1.16 (3H, d, 6.5)	14.0
20	1.22 (3H, d, 6.6)	18.1	1.33 (3H, d, 6.5)	18.4
21-NH	9.85 (1H, s)		9.81 (1H, s)	
22	8.34 (1H, s)	160.8	8.32 (1H, s)	160.7
1'		175.9		
2'	2.68 (1H, m)			
3'	1.16 (3H, d, 7.2)	19.1		
4'	1.16 (3H, d, 7.2)	19.1		
8-OH			5.66 (1H, d, 8.0)	
13-OH	12.70 (1H, bs)		12.68 (1H, bs)	

Table S2. Cytotoxicity of compounds **1**−**3** against human umbilical vein endothelial cells (HUVEC).

Compound	LD_{50} (μM)
**1**	62.6
**2**	34.6
**3**	192.9
